# Antimicrobial Activity of the Circular Bacteriocin AS-48 against Clinical Multidrug-Resistant *Staphylococcus aureus*

**DOI:** 10.3390/antibiotics10080925

**Published:** 2021-07-30

**Authors:** Cristina Velázquez-Suárez, Rubén Cebrián, Carmen Gasca-Capote, Antonio Sorlózano-Puerto, José Gutiérrez-Fernández, Manuel Martínez-Bueno, Mercedes Maqueda, Eva Valdivia

**Affiliations:** 1 Department of Microbiology, Faculty of Sciences, University of Granada, Av. Fuente Nueva s/n, 18071 Granada, Spain; cristina.velazquez@ibvf.csic.es (C.V.-S.); mmartine@ugr.es (M.M.-B.); mmaqueda@ugr.es (M.M.); evavm@ugr.es (E.V.); 2 Institute of Plant Biochemistry and Photosynthesis, CSIC, Universidad de Sevilla, Av. Américo Vespucio, 49, 41092 Seville, Spain; 3 Department of Molecular Genetics, Groningen Biomolecular Sciences and Biotechnology Institute, University of Groningen, Nijenborgh 7, 9747AG Groningen, The Netherlands; 4 Clinical Unit of Infectious Diseases, Microbiology and Preventive Medicine, Institute of Biomedicine of Seville (IBIS), Virgen del Rocío University Hospital, CSIC, University of Seville, Av. Manuel Siurot, s/n, 41013 Seville, Spain; mcgasca-ibis@us.es; 5 Department of Microbiology, School of Medicine and PhD Program in Clinical Medicine and Public Health, University of Granada, Avda. de la Investigación 11, 18016 Granada, Spain; asp@ugr.es (A.S.-P.); josegf@ugr.es (J.G.-F.); 6 Laboratory of Microbiology, Virgen de las Nieves University Hospital, Avda. de las Fuerzas Armadas 2, 18012 Granada, Spain

**Keywords:** *Staphylococcus aureus* MRSA, enterocin AS-48, antibiotic resistance, biofilms

## Abstract

The treatment and hospital-spread-control of methicillin-resistant *Staphylococcus aureus* (MRSA) is an important challenge since these bacteria are involved in a considerable number of nosocomial infections that are difficult to treat and produce prolonged hospitalization, thus also increasing the risk of death. In fact, MRSA strains are frequently resistant to all β-lactam antibiotics, and co-resistances with other drugs such as macrolides, aminoglycosides, and lincosamides are usually reported, limiting the therapeutical options. To this must be added that the ability of these bacteria to form biofilms on hospital surfaces and devices confer high antibiotic resistance and favors horizontal gene transfer of genetic-resistant mobile elements, the spreading of infections, and relapses. Here, we genotypically and phenotypically characterized 100 clinically isolated *S. aureus* for their resistance to 18 antibiotics (33% of them were OXA resistant MRSA) and ability to form biofilms. From them, we selected 48 strains on the basis on genotype group, antimicrobial-resistance profile, and existing OXA resistance to be assayed against bacteriocin AS-48. The results showed that AS-48 was active against all strains, regardless of their clinical source, genotype, antimicrobial resistance profile, or biofilm formation capacity, and this activity was enhanced in the presence of the antimicrobial peptide lysozyme. Finally, we explored the effect of AS-48 on formed *S. aureus* biofilms, observing a reduction in *S. aureus* S-33 viability. Changes in the matrix structure of the biofilms as well as in the cell division process were observed with scanning electron microscopy in both S-33 and S-48 *S. aureus* strains.

## 1. Introduction

*Staphylococcus aureus* is an important pathogen that causes a wide variety of diseases in humans and animals that range from skin or soft tissue infections to systemic and often fatal diseases [1]. This scenario is exacerbated by the ability of this bacterium to form biofilms on many surfaces, which increase staphylococci mutability, accelerating the emergence of antibiotic resistance through horizontal gene transfer [2,3,4]. According to the antimicrobial resistance in the EU/EEA report (EARS-Net), this bacterium was responsible for 20.6% of infections in 2019 [5] and as occurs with other pathogens, the increasing number of antibiotic-resistant *S. aureus* is seriously impairing the ability to control this pathogen, especially in patients in intensive care units (ICU), connected to a ventilator, or after surgical processing [6]. To this, the epidemic spread of methicillin/oxacillin resistant *S. aureus* (MRSA) must be added, which although currently showing decreasing trends in Europe [5], remains an important pathogen being the most common cause of bloodstream infections and exhibits a high burden in terms of morbidity and mortality [7]. Infections caused by MRSA are of special interest due to the difficulty of their treatment. In fact, MRSA strains are responsible for 10 times more infections than all multi-drug resistant (MDR) Gram-negative pathogens combined [8]. These strains are resistant to all ß-lactam antibiotics including cephalosporins and carbapenems, and are considered the prototype of multi-resistant nosocomial pathogens [9]. In addition, hospital-associated MRSA isolates are often multi-resistant to other commonly used antimicrobial agents such as erythromycin, clindamycin, and tetracycline [10,11]. Based on this, it is unsurprising that MRSA is in the group of pathogens for which new antimicrobials are urgently required according to the WHO priority list [12].

An emergent strategy for fighting bacterial infections produced by antibiotic-resistant organisms is based on the use of antimicrobial peptides (AMPs) [13]. AMPs are peptides that generally have a cationic amphiphilic nature and are produced by a wide variety of organisms, animals (including humans), plants, fungi, and bacteria where they are the first line of defense [14,15,16]. AMPs exert their antimicrobial effects by interacting with the bacterial cell membrane, which destabilizes and permeabilizes without harming eukaryotic cells. This selective antibacterial activity is likely due to the fact that the positive charge (s) on the α-helix surface of AMPs interact with negatively charged bacterial membranes, while the eukaryotic cell membranes are composed of uncharged neutral lipids [17]. AMPs produced by bacteria are called bacteriocins. Bacteriocins can be defined as ribosomally synthesized antimicrobial peptides produced by bacteria that are active against more or less closely related bacteria, either in the same species (narrow-spectrum) or across genera (broad-spectrum) [18]. The enterocin AS-48 is a circular bacteriocin (head-to-tail linked) produced by *Enterococcus* strains formed of 70 amino acid residues and of cationic-amphiphilic nature [19]. AS-48 has a potent antibacterial activity alone or in combination with other drugs against both Gram-positive and Gram-negative pathogens and even some parasites (*Trypanosomatidae*) [20,21,22,23,24,25]. Interestingly, AS-48 showed no hemolytic activity or toxicity against several cell lines and no toxic effect was observed in mice after the intraperitoneal or oral administration of the bacteriocin, suggesting the biosafety of this peptide [26,27].

In this work, we isolated and characterized from an antibiotic-resistant profile perspective 100 *S. aureus* from different health care units at the University Hospital of Granada (Spain). After that, based on the phenotypic antibiotic-resistant profile and genotype, 48 strains were selected to study the effect of the bacteriocin AS-48. Finally, to increase the inhibitor activity of AS-48, we combined the bacteriocin with a natural antimicrobial compound the lysozyme. The effect of AS-48 on formed biofilms of *S. aureus* was also studied by scanning electron microscopy.

## 2. Results and Discussion

### 2.1. Sample Collection and Identification

**Figure 1:**
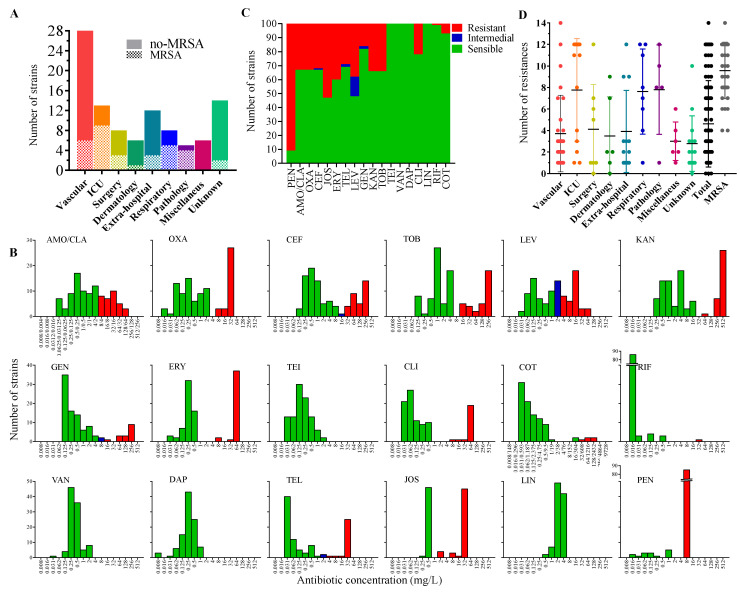
Antibiotic-resistance profile of the *S.* *aureus* strains. (**A**) Percentage of the *S. aureus* isolated in different hospital units. (**B**) *S. aureus* strains MIC distribution for the 18 antibiotics tested. The MICs range for sensitive strains is in green, intermedial in blue, and resistant strains in red. (**C**) The proportion of resistant, sensitive, and/or intermedial strains for each antibiotic tested. (**D**) Number of resistance identity by strain and medical care unit. Each dot represents one strain. MRSA indicates the number of resistances in OXA resistant strains. Miscellaneous represents a group of samples isolated from different hospital-care units such as Oncology (2), Rheumatology (1), Infectious Diseases (1), Pediatric (1), and Otorhinolaryngology (1).

One hundred strains of Gram-positive coccus were collected by the Microbiology Service of the University Hospital San Cecilio in Granada, Spain from several clinical departments (Figure 1A). They were initially characterized and identified as *S. aureus* using a Wider identification system [28]. The strains were genotyped by RAPD-PCR using the M13 primer and assigned to 21 genotypes (Supplementary Table S1) based on Pearson’s correlation coefficient and a similarity threshold of 80%. Representative candidates of each group were selected for 16s RNA amplification and sequencing analysis, confirming the strains as *S. aureus* at 99–100% identity.

### 2.2. Antimicrobial-Resistance Profile of the Isolates

**Figure 2:**
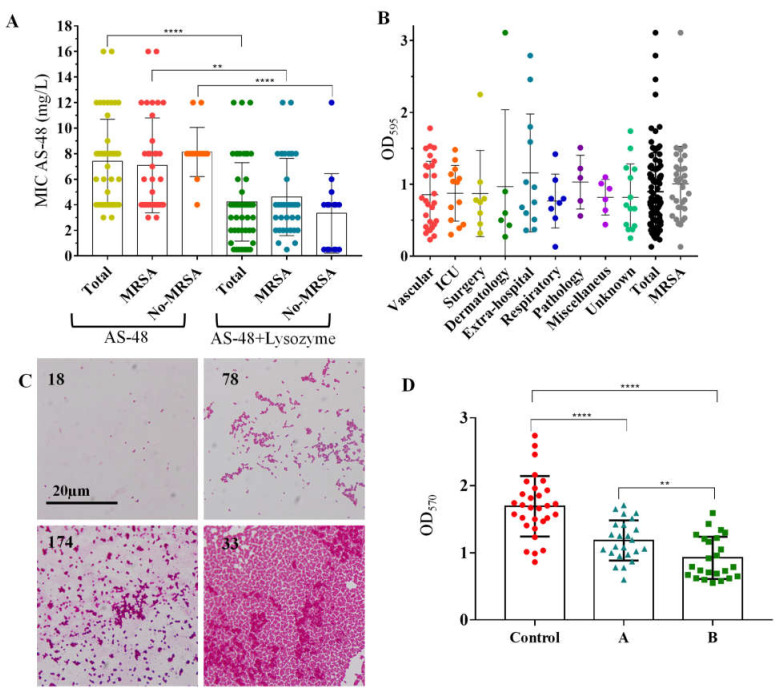
**(A**) Antimicrobial activity of AS-48 alone or in combination with lysozyme against the clinical *S. aureus* isolates. Each dot represents one strain. **** Significant differences at *p* < 0.0001. ** Significant differences at *p* = 0.0032 (**B**) Evaluation of biofilm formation for the different isolates according to their isolation source. Each dot represents one strain. Total indicates these values for all strains and MRS for the OXA positive isolates (**C**) Optical microscopy of stained biofilms formed by strains representative of the different biofilm formation capacities (OD_595_). Strain 18 OD_595_ = 0.13, strain 78 OD_595_ = 0.87, strain 174 OD_595_ = 1.51, and strain 33 OD_595_ = 3.11. (**D**) Antimicrobial activity of AS-48 on *S. aureus* 33 biofilms. A shows cell viability after 48 h in the presence of 32 mg/L AS-48 when kept unrenewed and B shows the cell viability of biofilms when washed after 24 h of treatment and added AS-48 again at the same concentration. The dots, triangles, and squares represent replicates for each of the indicated treatments or control. **** Significant differences at *p* < 0.0001. ** Significant differences at *p* = 0.0051.

All the isolates were tested against a panel of 18 antibiotics in a WIDER I system. Figure 1B shows the MIC distribution profile for these antibiotics as well as the number of resistant strains observed for the *S. aureus* collection in Figure 1C. As expected, almost all the strains (90%) were penicillin (PEN)-resistant (this resistance emerged shortly after the antibiotic’s introduction in the early 1940s) [29] while the average resistance to other β-lactam-related antibiotics used such as amoxicillin/clavulanic acid (AMO/CLA) and cefazolin (CEF) was the same as for oxacillin (OXA) (33%), since as mentioned in the introduction, resistance to this antibiotic (methicillin) is related to strong resistance to other β-lactam-related antibiotics [9]. This value is considerably higher than the Spanish average for MRSA bacteria, which was 19.2% of isolates in 2019 [5]. Regarding macrolide related antibiotics, higher resistances were observed for josamycin (JOS) and erythromycin (ERY) (53–40% of strains respectively) while 29% of the isolates were sensitive to telithromycin (TEL) (Figure 1C). For aminoglycosides, the observed resistance ranged between 16% of the tested strains for gentamicin (GEN) and 34% for tobramycin (TOB) and kanamycin (KAN) (Figure 1C). Finally, 22%, 7%, and 1% of the strains were clindamycin (CLI), cotrimoxazole (COT), and rifampicin (RIF) resistant (Figure 2C). All strains were also sensitive to the glycopeptides vancomycin (VAN) and teicoplanin (TEI) as well as the lipopeptides daptomycin (DAP) and the lincosamide clindamycin (CLI) (Figure 1C).

Considering the health care source of the isolates, the highest resistance levels were observed for strains isolated from the ICU, General-Pathology- and Respiratory-related units, while the lowest resistance levels were observed in strains isolated in Vascular- and Dermatology-related care units (Figure 1D). Strains isolated from these health care units were also the most frequent MRSA (Figure 1A). In fact, ICU patients and intubated patients are usually the most susceptible to *S. aureus* infections [6]. Besides, ICUs are often considered the epicenter for the development, amplification, and dissemination of drug-resistant microorganisms because of the invasive procedures that compromise the anatomical barriers, which could be related to the high resistance levels observed for the strains isolated at this care unit or related care unit [30]. The average antibiotic resistance observed in the full collection was about 5/16 (Table 1). The average antibiotic-resistances observed for the strains isolated in ICUs and General-Pathology- and Respiratory-related units were about 8/16 antibiotic tested while for the remainder, it was around 4/16. Overall, from the 100 strains isolated, three were not-resistant to any of the tested antibiotics, 27 to one antibiotic, 14 to two, 13 to three, six strains to four antibiotics, five to seven, five to eight, three to nine antibiotics, three to 10, three strains to 11 antibiotics, 11 to 12, and one to 14 antibiotics (Table 1, Figure 1D). The antibiotic-resistance profile for the different isolates is listed in Table 1. The most abundant antibiotic-resistance profile was the resistance to PEN only (27% of the isolates), followed by PEN, AMO/CLA, OXA, CEF, GEN, KAN, TOB, levofloxacin (LEV), TEL, ERY, JOS, CLI resistant profile (9% of isolated), and resistance to PEN, JOS, or PEN, KAN, TOB, which was 5% of the cases (Table 1). One strain was resistant to 14/18 of the antibiotics tested (PEN, AMO/CLA, OXA, CEF, GEN, KAN, TOB, LEV, TEL, ERY, JOS, CLI, COT + RIF) (Table 1). In total, 41 antibiotic-resistant groups were observed for the bacterial collection. Regarding MRSA strains, it has been referred that in addition to methicillin/oxacillin, they are resistant to most of the common clinically used antimicrobial agents including other β-lactams, aminoglycosides, macrolides, and clindamycin among others [31,32,33,34]. Co-resistance to all the β-lactams and macrolides was observed in 6% of the strains, co-resistance to all β-lactam and aminoglycosides in 2%, and co-resistance to the antibiotics tested on these three antibiotic groups was detected in 11% of the samples (Table 1). Co-resistance to all β-lactams and CLI was observed in 17% of the cases (Table 1).

### 2.3. Antimicrobial Activity of AS-48 Alone and in Combination with Lysozyme

Based on the antimicrobial-resistance profile and considering the existing OXA resistance (MRSA) and the genotyped groups, 48 strains (Table 1 and Table S1) were selected for further analysis, exploring the antimicrobial activity of AS-48 alone. As shown in Figure 2A, almost all strains were sensitive to AS-48 at concentrations ranging from 3 to 16 mg/L (Table S1) with an average resistance of 7.4 ± 0.46 mg/L, suggesting that the AS-48 activity is independent of the antimicrobial-resistance profile or genotype. This MIC was 4.5 times higher than the average MIC observed for food-isolated *S. aureus* (1.63 ± 0.06 mg/L) [21], which could also be related to the pathogeny of these clinical isolates. No significant differences (*p* = 0.3078) were observed between MRSA and non-MRSA strains regarding the AS-48 sensitivity, although MRSA strains were slightly more sensitive to AS-48 than no-MRSA strains (7.086 ± 0.62 vs. 8.13 ± 0.49 mg/L, respectively) (Figure 2A).

Next, we analyzed the antimicrobial activity of AS-48 combined with lysozyme (at 4 g/L), a natural antimicrobial peptide that is part of the innate immune system of animals and which is highly localized in the lungs, where *S. aureus* is an important pathogen [35]. The combination of AS-48 with lysozyme has shown strong synergism in the past against other pathogens such as *Cutibacterium acnes* or *Mycobacterium tuberculosis* [20,36]. In this case, and despite *S. aureus* being naturally resistant to lysozyme due to the O-acetylation of the *N*-acetylmuramic acid in peptidoglycan, significant differences were observed for the AS-48 MIC in the presence of lysozyme, reducing the average MIC of these strains to 4.23 ± 0.43 mg/L (*p* < 0.0001) with a MIC ranging from 0.5 to 12 mg/L (Figure 2A). The mechanistic basis of this effect (considering that *S. aureus* is lysozyme resistant) is unclear. The potentiation of AS-48 activity by lysozyme was studied for the first time by Ananou et al. (2018) against food *S. aureus* strains [37]. It was hypothesized that this might be explained by autolysis triggered by the enterocin in this bacterium from the first minutes of contact. This AS-48-induced autolysis could render the cell wall more susceptible to the action of the muramidase on other putative lysozyme targets, different to the glycosidic β-1,4 bond between *N*-acetylmuramic acid and *N*-acetylglucosamine [37]. As for the treatment with AS-48 alone, no significant differences in the MIC were observed for non-MRSA strains (3.33 ± 0.80 mg/L) and MRSA strains (4.61 ± 0.51 mg/L, *p* = 0.1796) (Figure 2A). When comparing the MIC between groups according to the presence or absence of lysozyme, significant differences were observed in both cases for MRSA (*p* = 0.0032) and no-MRSA (*p* < 0.0001) (Figure 2A). The individual MICs for each tested strain are listed in Supplementary Table S1.

### 2.4. Effect of AS-48 on S. aureus Biofilms

Biofilm growing bacteria are a paradigm in the treatment of infections since bacteria growing in these conditions dramatically increase the MIC for antibiotics, and *S. aureus* is not the exception [38,39]. Besides, *S. aureus* biofilms promote the horizontal transfer of antibiotic resistance. Together with a higher antibiotic tolerance due to the low antibiotic permeability into the biofilm, it makes this biological structure a real barrier for the treatment of bacteria [4]. Biofilms made up of staphylococci are frequently involved in skin and soft-tissue infections, endocarditis, urinary tract and bone-related infections, and implant-related infections [40]. To this, it must be added that the presence of persister cells inside the biofilms is also related to chronic and relapsing infections [41], so activity on biofilms is a desired characteristic when developing new drugs.

#### 2.4.1. Biofilms Formation by *S. aureus* Isolates

Prior to the study of the effect of AS-48 on *S. aureus* biofilms, we checked the ability of *S. aureus* strains to form biofilms using the method of Toledo-Arana et al., (2001), classifying the biofilm formation ability as weak, moderate, or strong [42]. Figure 2B shows the ability of each strain to form biofilms depending on the isolation source. A total of 62% of the strains had weak ability to form biofilms, 34% moderate ability, and 4% strong ability (Figure 2B). Figure 2C shows examples of the biofilm appearance using an optical microscope for different strains considering the ability to form them (strain 185 very weak, strain 78 weak to moderate, strain 174 moderate, strain 33 strong). Similar biofilm formation capacities have been observed for *S. aureus* collections in other studies [43]. Strain 33 isolated from the Dermatology Service was the strongest forming biofilms. It was also an MRSA strain with the phenotype resistant to PEN, AMO/CLA, OXA, CEF, KAN, TOB, LEV, ERY, and JOS, so we selected this strain for further studies.

#### 2.4.2. Effect of Bacteriocin AS-48 against *S. aureus* Biofilms

Different strategies have emerged to fight bacterial infections caused by biofilms focusing on each of the four stages of the biofilm lifecycle [44]: (1) prevention of bacterial adhesion; (2) weakening strategy to avoid the biofilm maturation; (3) disruption to promote biofilm dispersion of advanced stage biofilms; and (4) killing that corresponds to the eradication of a mature biofilm. The latter is the most complex stage, as mature biofilms are rather untreatable. The use of AMPs has been proposed to kill bacteria in mature biofilms and some bacteriocins have been suggested for the eradication of bacteria embedded in biofilms [45,46,47]. Considering that the target of bacteriocins is mainly the bacterial membrane (which is not prone to the development of resistance) and that the activity of bacteriocins is generally bactericidal, they could be an interesting alternative to current antibiotics for treating biofilm-related infections [48]. Regarding AS-48, anti-biofilm activity has previously been observed against *C. acnes* [20], so we tested its activity against strain 33, evaluating the viability of the cells and also analyzing the structure of the biofilm after the treatment using SEM. The MIC for this strain was 4 mg/L, but since cells in biofilms are more resistant, we treated formed biofilms with 32 mg/L of AS-48. To determine the cell viability, the treatment was prolonged for 48 h, adding AS-48 once (at the beginning) or twice (washing after 24 h and then adding AS-48 again at the same concentration). Afterward, we evaluated the cell viability following the method proposed by Sabaeifard et al. (2014) [49].

As shown in Figure 2D, the addition of 32 mg/L of AS-48 to *S. aureus* 33 biofilms significantly decreased the amount of formazan formed by the reduction of triphenyl tetrazolium chloride (TTC) by the cell biofilms, both when AS-48 was kept unrenewed for 48 h (*p* < 0.0001) and when it was washed after 24 h and added again for another 24 h (*p* < 0.0001). This second option was also statistically more effective than adding AS-48 only once (*p* = 0.0051) (Figure 2D). The decrease in formazan can be attributed to the direct bactericidal effect of AS-48 on staphylococci embedded in biofilms and also to the loss of biomass that may occur in biofilms when a great number of cells die due to the effects of antimicrobials and pieces of biomass formed (cells and matrix) break off from the biofilm.

#### 2.4.3. Ultrastructure of *S. aureus* Biofilms Treated with AS-48

**Figure 3:**
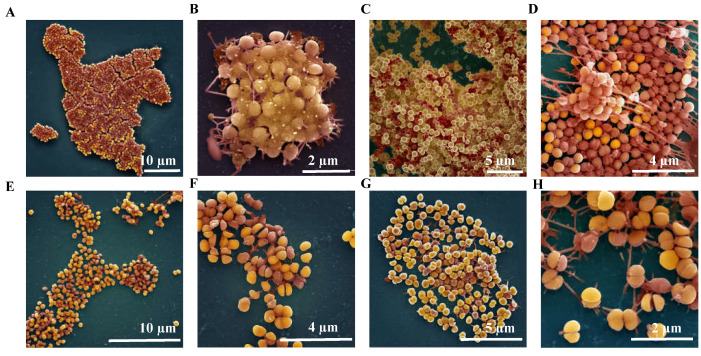
Biofilms of *S. aureus* 33 observed under scanning electron microscope. (**A**,**B**) Control biofilms formed after 24 h. (**C**,**D**) Control biofilms formed after 48 h. (**E**,**F**) Biofilms treated for 48 h with 16 µg/mL AS-48. (**G**,**H**) Biofilms treated for 48 h with 32 µg/mL.

To visualize the effects produced by bacteriocin AS-48 on staphylococcal biofilms using SEM, strain 33 was allowed to adhere to microslides for 24 h or 48 h before being treated with AS-48 (16 or 32 mg/L) for 24 h and then processed to be observed by SEM. Figure 3 shows the microphotographs of the biofilms of strain 33. Figure 3A–D corresponds to control non-AS-48-treated biofilms (after 24 h Figure 3A,B and 48 h Figure 3C,D) and as expected for a system severely limited by nutrients, the biofilm was formed of discrete microcolonies (Figure 3A,B). These microcolonies were densely covered by a matrix where staphylococcal cells were imbibed. This matrix formed was filamentous, anchoring the biofilm to the substrate. In fact, the cords of the matrix can be seen coming out of the cells and connecting cell to cell and cells with the substrate. In Figure 3C,D, where the biofilm had grown for 48 h, a greater tridimensionality was observed. The treatment with AS-48 for 24 h produced significant changes in biofilms at both concentrations, 16 mg/L (Figure 3E,F) and 32 mg/L of bacteriocin (Figure 3G,H), losing almost all the matrix. This essential component of the biofilm had almost disappeared, although some filaments anchoring the cells to the substrate were visible (Figure 3G,H). As a consequence of the lack of matrix, the cell surface was rather smooth, in contrast to the rough surface of many of the cells in untreated biofilms.

One surprising morphologic change observed in the AS-48-treated biofilms is the existence of numerous cells with division septa, the septum being completed in many of them, although the cells remained together. A similar phenotype has been previously described for other AMPs such as the OaBac5mini peptide, an ovine-derived antimicrobial peptide [50]. These authors suggested that this could be related to the cell division process stopping at this point, whereas the control cells moved on from this state and continued to divide as normal. To exert this action, it has been proposed that peptides must have another intracellular non-membrane target and therefore must cross the cytoplasmic membrane to translocate the cytoplasm [51]. Regarding AS-48, alternative targets are still unclear, although transcriptomic data obtained from AS-48-treated *Bacillus cereus* cells suggest this possibility [52]. Besides, it has recently been described that AS-48 is active against intracellular pathogens not altering or killing the eukaryotic host cell. This data suggest that AS-48 could act as a cell-penetrating peptide, proposing that its target might not be restricted to the bacterial membranes [24,36,53]. Similar morphologic changes were also observed in the cell biofilm of strain 48 (a good biofilm former) after AS-48 treatment (Supplementary Figure S1).

## 3. Materials and Methods

### 3.1. Bacterial Isolation and Growth Conditions

A total of 100 Gram-positive coccus strains isolated from clinical cases in different health care services at the Microbiology Laboratory of San Cecilio University Hospital in Granada, Spain were used in this study. Twenty-seven samples were isolated from the Cardiology/Vascular service, 13 samples from the intensive care unit (ICU), eight samples from the Surgery service, six samples from the Dermatology service, eight samples from the Respiratory service, five samples from the General Pathology service, 12 samples were obtained from extra-hospital sources (samples sent by family doctor), 14 samples were isolated from an unknown hospital source, two samples from the Oncology service, one from Rheumatology, one from the Infectious Diseases service, one from the Pediatric service, and another from the Otorhinolaryngology service. Bacteria were routinely cultured in tryptic soy broth (TSB, Scharlau, Barcelona, Spain) or tryptic soy agar (TSA, Scharlau) and identified as *S. aureus* using a Wider I system (Francisco Soria Melguizo SA, Madrid, Spain).

### 3.2. Genotyping and Molecular Identification: RAPD-Analysis and 16S rDNA Gene Sequencing

Genomic typing of the clinical isolates staphylococci was initially performed by randomly amplified polymorphic DNA (RAPD) followed by the 16s rDNA amplification and sequencing of representative members of each group. For this, genomic DNA was isolated according to the modified salting out procedure described in Martín-Platero et al. (2007) [54]. The genotypic fingerprint for the strains was obtained by the amplification with the M13 as a primer (5′-GAGGGTGGCGGTTCT-3′) as described previously [55]. Next, the genomic profiles were clustered using the Fingerprinting II software (Bio-Rad) and a similarity matrix was constructed based on the Pearson correlation coefficient, and the corresponding dendrogram was deduced using the unweighted pair group method (UPMGA) with arithmetic averages. The level of identity for strain discrimination was set at 80% similarity for different genotypes. Finally, the 16s rDNA of a representative sample of each genomic cluster was amplified with the primers WO1 (5′-AGAGTTTGATC[AC]TGGCTC-3′) and WO12 (5′-TACGCATTTCACC[GT]CTACA-3′) [56]. PCR products were purified with an Accuprep PCR Purification Kit (Bioneer, Daejeon, Korea) and were sequenced at the Center for Scientific Instrumentation of the University of Granada using an ABI Prism dye terminator cycle sequencing ready-reaction automated sequencer (ABI 3100; Applied Biosystems, Madrid, Spain). Homologies were searched for on the BLASTN database (National Center for Biotechnology Information) using BLAST.

### 3.3. Enterocin AS-48 Purification

AS-48 was purified from cultures of the *Enterococcus faecalis* UGRA10 strain [57] in Esprion 300 plus glucose (1%) (DMV Int., Veghel, The Netherlands) in a pH-controlled device under the conditions previously established [58]. Briefly, the fermented supernatant was purified by cationic exchange chromatography on a carboxymethyl sepharose matrix (CM25, GE Amersham) and desalted and concentrated using reverse-phase chromatography on C18 silica beads (Water). The bacteriocin was purified to homogeneity by RP-HPLC. The protein concentration was determined by measuring the UV absorption at 280 nm in a Nanodrop 2000 (Thermo Scientific, St. Louis, MI, USA).

### 3.4. Determination of the Minimal Inhibitory Concentration (MIC)

A WIDER I system was used to check the antibiotic sensitivity of the clinical isolates against 18 different antibiotics as penicillin B (PEN), amoxicillin/clavulanic acid (AMO/CLA), oxacillin (OXA), cefazolin (CEF), gentamicin (GEN), tobramycin (TOB), kanamycin (KAN), erythromycin (ERY), telithromycin (TEL), josamycin (JOS), levofloxacin (LEV), vancomycin (VAN), teicoplanin (TEI), daptomycin (DAP), clindamycin (CLI), rifampicin (RIF), cotrimoxazole (COT), and linezolid (LIN) [59]. The bacteriocin AS-48 was assayed in Mueller-Hinton broth alone or combined with lysozyme (4 mg/mL, Amresco) in 96-well polystyrene plates following the microdilution method described by the Clinical and Laboratory Standards Institute [60]. Considering the previously established susceptibility of staphylococci to AS-48 [21,61], the bacteriocin concentrations used ranged from 0.5 to 32 mg/L.

### 3.5. Antimicrobial Activity of AS-48 on S. aureus Biofilms

#### 3.5.1. Biofilm Formation by the *Staphylococci*

First, the ability of the isolated strains to form biofilms was tested as described by Toledo-Arana et al. (2001) [42], with some modifications. Briefly, a 16–18 h culture of the desired strain in BHI was diluted (1/40) in the same medium and 200 µL of this bacterial suspension were poured into the microplate wells. Then, the plate was incubated for 24 h at 37 °C and afterward, the liquid of the wells was removed and the wells were washed twice with phosphate buffer saline (PBS). Next, the wells were left to dry in the inverted position and then dyed for 15 min with crystal violet solution (1 g crystal violet dissolved in a mixture of 80 mL distilled water plus 20 mL 96% ethanol). Then, the wells were washed four more times and finally, the crystal violet absorbed by the proteins and DNA of cells in biofilm was extracted with 200 µL ethanol 96%. The crystal violet extracted from cells was quantified by measuring the OD_595_ of the ethanolic extract in the wells in a Tecan Sunrise *X*-Fluor 4 microplate reader. Each strain was assayed in triplicate and the test was repeated twice. Biofilm formation was evaluated as: very weak or no biofilm producers (0< OD_595_< 0.5), weak (0.5 ≤ OD_595_ < 1), moderate (1 ≤ OD_595_ < 2), and strong (OD_595_ ≥ 2) [42].

#### 3.5.2. Visualization under the Scanning Electron Microscope (SEM) of Effects Caused by AS-48 on *S. aureus* Biofilms

Before biofilm observation by SEM, the conditions of biofilm formation in conventional slides were established and visualized under the optical microscope. For optical microscope observation, slides (75 × 25 mm) were immersed in 50 mL Falcon tubes containing 35 mL sterile saline solution (0.85% NaCl), and then inoculated with 0.5 mL of a 24 h culture of each staphylococcal strain and then incubated at 37 °C for 24 h. The biofilms formed were heat-fixed, stained with fuchsine, and observed under a microscope Olympus BX51 microscope assisted with an Olympus DP72 camera.

For SEM observation, *S. aureus* biofilms were formed on 10 × 10 mm slides of bioactive glass (BonAlive1 Biomaterials LTD, Turku, Finland). Slides were immersed in Eppendorf tubes containing 1 mL sterile saline solution (0.85% NaCl), inoculated with 14 μL of a 24-h culture of the selected staphylococcal strain, incubated for 24 or 48 h at 37 °C, and then treated with AS-48 at 16 or 32 mg/L for 24 h at 37 °C. Control untreated biofilms were also established. Afterward, the slides were removed, rinsed with sterile saline solution and pre-fixed with a 2.5% (*v*/*v*) glutaraldehyde (Merck, Madrid, Spain) solution in 0.1 M sodium cacodylate buffer, pH 7.2 at 4 °C for 2 h, followed by three washes in the same cacodylate buffer. Fixed samples were prepared for electron microscopy examination at the Center for Scientific Instrumentation at the University of Granada. Samples were also postfixed with 1% osmium tetroxide in the same buffer at 4 °C for 1 h, dehydrated by immersion in a graded series of ethanol and dried by critical point drying in a Leica EM CPD300 dryer (Wetzlar, Germany). Afterward, the specimens were coated with a layer of carbon in a thermal evaporator EMITECH K975X (Quorum Technologies, Laughton, England) and then observed in a high-resolution environmental field emission scanning electron microscope with quemscan and cryoestation (model QuemScan 650F, Thermo Fisher Scientific, St. Louis, MI, USA).

#### 3.5.3. Studies of the Effects Caused by AS-48 on the Viability of *S. aureus* Cells Imbibed in Biofilms

To find out the residual cellular viability in biofilms after treatment with bacteriocin, the method of Sabaeifard et al. (2014) was followed with some modifications [49]. For biofilm formation, staphylococci were grown in a 96-well plate for 24 h and then AS-48 was added to a final concentration of 32 mg/L. The bacteriocin was maintained for 48 h with or without bacteriocin renewal at 24 h. After 48 h, the medium was removed and 150 μL TSB plus 0.2% glucose and 50 μL triphenyl tetrazolium chloride (1% TTC sterile solution, Scharlau) were added and incubated at 37 °C for an additional 4 h. One hundred microliters of solubilization medium were then added in order to extract the red dye (formazan) formed by the reducing activity of viable staphylococci and the plate was incubated at 27 °C overnight under agitation. Finally, the supernatants were transferred to a new 96-well plate and the formazan produced from the TTC and solubilized by the formazan solubilization medium was measured spectrophotometrically at 570 nm in a Tecan Sunrise *X*-Fluor 4 microplate reader. Each experimental variant and the control were repeated eight times for the same plate and four independent replicas of the complete experiment were carried out. The composition of the formazan solubilization medium and the preparation procedure was as described by Riis et al. (2013) [62]: dimethylformamide was added (40%, *v:v*) to 2% (*v:v*) glacial acetic acid. Then, 16% (wt/vol) sodium dodecyl sulfate (SDS) was added to the above mixture and allowed to dissolve, and the pH was adjusted to 4.7. The reactive was stored at room temperature to avoid precipitation of SDS. If a precipitate formed, the reactive was warmed at 37 °C and mixed to solubilize SDS.

### 3.6. Statistical Analysis

Statistical analysis was performed using Graph-Pad Prism 8. All data were presented as means ± SD. Unpaired student’s *t*-test between two groups was used to calculate the *p*-values (* *p* <0.05, ** *p* < 0.01; *** *p* < 0.001; **** *p* < 0.0001; ns, not significant).

## 4. Conclusions

In this study, we analyzed the antibiotic-resistance profile of a collection of 100 *S. aureus* strains genotypically characterized in 21 groups to 18 antibiotics. These were isolated from different health care units at the University Hospital of Granada, Spain. Thirty-three percent of these strains were phenotypically MRSA, and co-resistance of these strains to other antibiotics such as aminoglycosides, macrolides, or clindamycin was broadly observed in the collection, regardless of the strain genotype. However, strains isolated in ICU and Respiratory and General Pathology services were the most frequent MRSA and had higher levels of co-resistance to other antibiotics. Irrespective of the genotype or the antibiotic-resistance profile, all the strains were sensitive to AS-48 at an average concentration of 7.4 ± 0.46 mg/L. This activity was enhanced by lysozyme (4.23 ± 0.43 mg/L), despite *S. aureus* being resistant to this antimicrobial. Finally, we analyzed the biofilm formation capacity of the strains, and the best were selected to test the activity of AS-48 against *S. aureus* embedded in the biofilms. Interestingly, AS-48 was also able to significantly kill cells growing on biofilms, inducing morphological changes that affected the matrix structure of the biofilm as well as the cell shape.

## Figures and Tables

**Table 1:** Antibiotic-resistant phenotypes for the different isolates. In bold, the strains selected for further analysis.

Phenotype	Strain Numbers	Origin (Number of Strains)
No antibiotic resistance	32, 72, 110	Surgery (1), Dermatology (1), Unknown (1)
PEN	4, 5, 6, 13, **21**, 24, 35, **47**, **48**, 52, 55, 73, **81**, 84, 85, 91, **97**, 100, 101, 106, 120, 147, 160, 191, 207, **208**, 221,	Vascular (10), Extra-hospital (4), Unknown (4), Surgery (3), ICU (2), Dermatology (1), Pathology (1), Miscellaneous (1)
LEV	207	Respiratory
PEN, JOS	77, **113**, 170, 190, **192**	Vascular (2), Dermatology (1), Extra-hospital (2), Miscellaneous (1)
PEN, TEL	3, 62, **68**, **105**	Unknown (2), ICU (1), Miscellaneous (1)
PEN, ERY	23, 122	Vascular
PEN, LEV	193	Unknown
PEN, COT	49	Extra-hospital
LEV, JOS	**12**	Dermatology
PEN, KAN, TOB	**14**, 142, 148, 205, 220	Extra-hospital (2), Vascular (1), ICU (1)
PEN, ERY, JOS	76, 114, 175, 214,	Vascular (2), Miscellaneous (1), Unknown (1)
PEN, TEL, JOS	118, 141	Unknown
PEN, LEV, JOS	218	Vascular
ERY, JOS, COT	29	Vascular
PEN, ERY, JOS, CLIN	16	Unknown
PEN, AMO/CLA, OXA, LEV	**78**	Vascular
PEN, AMO/CLA, OXA, CEF	**145**	ICU
PEN, KAN, TOB, TEL	153	Miscellaneous
GEN, KAN, TOB, COT	53	Vascular
LEV, ERY, JOS, CLIN	203	Respiratory
PEN, TEL, ERY, JOS, CLIN	103	Surgery
PEN, GEN, KAN, TOB, JOS	121	Vascular
PEN, AMO/CLA, OXA, CEF, LEV, JOS	**19**, **135**, **185**	Unknown (1), Surgery (1), Respiratory (1)
PEN, KAN, TOB, TEL, JOS, ERY	**75**	Miscellaneous
PEN, AMO/CLA, OXA, CEF, LEV, ERY, JOS	**20**, **87**	Surgery, vascular
PEN, LEV, TEL, ERY, JOS, CLI, COT	**28**	Vascular
PEN, GEN, KAN, TOB, TEL, ERY, JOS	79	Respiratory
KAN, TOB, LEV, TEL, ERY, JOS, CLI	139	Dermatology
PEN, AMO/CLA, OXA, CEF, LEV, TEL, ERY, JOS	**2**	Vascular
PEN, AMO/CLA, OXA, CEF, KAN, TOB, LEV, JOS	**119**, **204**	Pathology, Respiratory
PEN, AMO/CLA, OXA, CEF, LEV, ERY, JOS, CLI	**174**, **186**	Pathology, ICU
PEN, AMO/CLA, OXA, CEF, KAN, TOB, LEV, ERY, JOS	**33**, **154**, **171**	Extra-hospital (2), Dermatology (1)
PEN, AMO/CLA, OXA, CEF, GEN, KAN, TOB, ERY, JOS, CLI	**90**	Pathology
PEN, AMO/CLA, OXA, CEF, GEN, KAN, TOB, LEV, ERY, JOS	**176**	Vascular
PEN, AMO/CLA, OXA, CEF, KAN, TOB, LEV, TEL, ERY, JOS	**215**	Unknown
PEN, AMO/CLA, OXA, CEF, KAN, TOB, LEV, TEL, ERY, JOS, CLI	**1**, **152**, **155**,	ICU (2), Respiratory (1)
PEN, AMO/CLA, OXA, CEF, GEN, KAN, TOB, LEV, TEL, ERY, JOS, CLI	**17**, **30**, 80, **96**, **104**, **111**, **112**, **136**, **219**	ICU (5), Respiratory (1), Vascular (1), Surgery (1), Pathology (1)
PEN, AMO/CLA, OXA, CEF, GEN, KAN, TOB, LEV, TEL, ERY, JOS, COT	**95**	Extra-hospital
PEN, AMO/CLA, OXA, CEF, KAN, TOB, LEV, TEL, ERY, JOS, CLI, COT	**54**	Respiratory
PEN, AMO/CLA, OXA, CEF, GEN, KAN, TOB, LEV, TEL, ERY, JOS, COT	**80**	Extra-hospital
PEN, AMO/CLA, OXA, CEF, GEN, KAN, TOB, LEV, TEL, ERY, JOS, CLI, COT, RIF	**94**	Vascular

## Data Availability

The data presented in this study are available in Supplementary Material.

## Supplementary Materials

The following are available online at https://www.mdpi.com/article/10.3390/antibiotics10080925/s1, Figure S1: Biofilms of *S. aureus* 48 observed under scanning electron microscope. (A,B) Control biofilms formed for 24 h. (C,D) Biofilms formed for 24 h treated with 16 µg/mL AS-48. Table S1: Summary table of the different parameters evaluated. Genomic profile aggrupation of the isolated strains indicating the isolation source, antibiotic-resistance profile, biofilm formation capacity, and the antimicrobial activity of AS-48 alone and in combination with lysozyme for selected strains of each group. In shadow, the MRSA strains.
