# Supplementary material for: Antimicrobial Activity of the Circular Bacteriocin AS-48 against Clinical Multidrug-Resistant Staphylococcus aureus
Source: Antibiotics (Basel). 2021 Jul 30;10(8):925. doi: 10.3390/antibiotics10080925 (PMC8388780; doi:10.3390/antibiotics10080925)
Supplement: Supplementary file 1 [file antibiotics-10-00925-s001.zip › antibiotics-1285030-supplementary/antibiotics-1285030-supplementary-final/Suppl. Table 1.pdf]

antibiotics

MDPI

Article

Antimicrobial Activity of the Circular Bacteriocin AS-48 against Clinical Multidrug-Resistant *Staphylococcus aureus*

Cristina Velázquez-Suárez [1,2], Rubén Cebrián [3,*], Carmen Gasca-Capote [4], Antonio Sorlózano-Puerto [5,6], José Gutiérrez-Fernández [5,6], Manuel Martínez-Bueno [1], Mercedes Maqueda [1] and Eva Valdivia [1]

1 Department of Microbiology, Faculty of Sciences, University of Granada, Av. Fuente Nueva s/n, 18071 Granada, Spain; cristina.velazquez@ibvf.csic.es (C.V.-S.); mmartine@ugr.es (M.M.-B.); mmaqueda@ugr.es (M.M.); evavm@ugr.es (E.V.)
2 Institute of Plant Biochemistry and Photosynthesis, CSIC, Universidad de Sevilla, Av. Américo Vespucio, 49, 41092 Seville, Spain
3 Department of Molecular Genetics, Groningen Biomolecular Sciences and Biotechnology Institute, University of Groningen, Nijenborgh 7, 9747AG Groningen, The Netherlands
4 Clinical Unit of Infectious Diseases, Microbiology and Preventive Medicine, Institute of Biomedicine of Seville (IBIS), Virgen del Rocío University Hospital, CSIC, University of Seville, Av. Manuel Siurot, s/n, 41013 Seville, Spain; mcgasca-ibis@us.es
5 Department of Microbiology, School of Medicine and PhD Program in Clinical Medicine and Public Health, University of Granada, Avda. de la Investigación 11, 18016 Granada, Spain; asp@ugr.es (A.S.-P.); josegf@ugr.es (J.G.-F.)
6 Laboratory of Microbiology, Virgen de las Nieves University Hospital, Avda. de las Fuerzas Armadas 2, 18012 Granada, Spain
* Correspondence: r.cebrian.castillo@rug.nl

Table S1. Summary table of the different parameters evaluated. Genomic profile aggrupation of the isolated strains indicating the isolation source, antibiotic-resistance profile, biofilm formation capacity, and the antimicrobial activity of AS-48 alone and in combination with lysozyme for selected strains of each group.

Genomic groups	Strains	Isolation source	Antibiotic resistances	AS-48	AS-48 +Lys	Biofilm
G-1	208	Surgery	PEN	4	2	2.25
	97	Unknown	PEN	12	8	0.37
	100	Dermatology	PEN	-	-	0.50
	145	ICU	PEN, AMO/CLA, OXA, CEF	4	2	0.44
	87	Vascular	PEN, AMO/CLA, OXA, CEF, LEV, ERY, JOS	8	8	1.12
G-2	91	Pathology	PEN	-	-	1.09
	78	Vascular	PEN, AMO/CLA, OXA, LEV	6	4	0.87
	79	Respiratory	PEN, GEN, KAN, TOB, TEL, ERY, JOS	-	-	0.80
G-3	101	Vascular	PEN	-	-	0.48
	120	Extra-hospital	PEN	-	-	2.46
	3	Unknown	PEN, TEL	-	-	0.42
	90	Pathology	PEN, AMO/CLA, OXA, CEF, GEN, KAN, TOB, ERY, JOS, CLIN	12	8	0.77
G-4	81	Vascular	PEN	6	8	0.36
G-5	21	ICU	PEN	8	0.5	0.39
	114	Vascular	PEN, ERY, JOS	-	-	0.40
	118	Unknown	PEN, TEL, JOS	-	-	0.44
	153	Miscellaneus	PEN, KAN, TOB, TEL	-	-	1.10
G-6	113	Vascular	PEN, JOS	8	4	0.28
G-7	192	Miscellaneus	PEN, JOS	4	2	0.44

**G-8**	77	Dermatology	PEN, JOS	-	-	0.90
	80	ICU	PEN, AMO/CLA, OXA, CEF, GEN, KAN, TOB, LEV, TEL, ERY, JOS, CLIN	4	4	1.21
	96	Respiratory	PEN, AMO/CLA, OXA, CEF, GEN, KAN, TOB, LEV, TEL, ERY, JOS, CLIN	4	3	1.42
	95	Extra-hospi-tal	PEN, AMO/CLA, OXA, CEF, GEN, KAN, TOB, LEV, TEL, ERY, JOS, COT	12	4	0.51
**G-9**	6	Unknown	PEN	-	-	1.50
	12	Dermatology	LEV, JOS	8	4	0.27
	23	Vascular	PEN, ERY	-	-	0.57
	16	Unknown	PEN, ERY, JOS, CLIN	-	-	0.25
	154	Extra-hospi-tal	PEN, AMO/CLA, OXA, CEF, KAN, TOB, LEV, ERY, JOS	4	1	0.96
	171	Extra-hospi-tal	PEN, AMO/CLA, OXA, CEF, KAN, TOB, LEV, ERY, JOS	4	3	0.68
	1	ICU	PEN, AMO/CLA, OXA, CEF, KAN, TOB, LEV, TEL, ERY, JOS, CLIN	8	4	1.34
	17	ICU	PEN, AMO/CLA, OXA, CEF, GEN, KAN, TOB, LEV, TEL, ERY, JOS, CLIN	8	0.5	1.03
**G-10**	28	Vascular	PEN, LEV, TEL, ERY, JOS, CLIN, COT	8	5	1.78
**G-11**	110	Unknown	No antibiotic resistance	-	-	1.74
	85	Vascular	PEN	-	-	0.36
	221	Vascular	PEN	-	-	0.49
	191	Miscellaneus	PEN	-	-	0.79
	106	Vascular	PEN	-	-	0.72
	4	Extra-hospi-tal	PEN	-	-	1.80
	147	ICU	PEN	-	-	0.30
	84	Vascular	PEN	-	-	0.81
	24	Extra-hospi-tal	PEN	-	-	0.37
	5	Vascular	PEN	-	-	0.77
	160	Surgery	PEN	-	-	0.75
	207	Respiratory	LEV	-	-	0.80
	193	Unknown	PEN, LEV	-	-	0.66
	170	Vascular	PEN, JOS	-	-	0.74
	190	Extra-hospi-tal	PEN, JOS	-	-	0.75
	122	Vascular	PEN, ERY	-	-	1.12
	220	Extra-hospi-tal	PEN, KAN, TOB	-	-	1.03
	205	Vascular	PEN, KAN, TOB	-	-	0.41
	148	Extra-hospi-tal	PEN, KAN, TOB	-	-	0.60
	142	Extra-hospi-tal	PEN, KAN, TOB	-	-	0.36
	218	Vascular	PEN, LEV, JOS	-	-	1.32
	214	Vascular	PEN, ERY, JOS	-	-	0.32
	175	Miscellaneus	PEN, ERY, JOS	-	-	0.94
	76	Unknown	PEN, ERY, JOS	-	-	1.23
	141	Unknown	PEN, TEL, JOS	-	-	1.09

	203	Respiratory	LEV, ERY, JOS, CLIN	-	-	0.74
	103	Surgery	PEN, TEL, ERY, JOS, CLIN	-	-	0.32
	121	Vascular	PEN, GEN, KAN, TOB, JOS	-	-	1.26
	185	Respiratory	PEN, AMO/CLA, OXA, CEF, LEV, JOS	4	2	0.13
	19	Unknown	PEN, AMO/CLA, OXA, CEF, LEV, JOS	12	0.5	0.82
	135	Surgery	PEN, AMO/CLA, OXA, CEF, LEV, JOS	8	4	0.78
	139	Dermatology	KAN, TOB, LEV, TEL, ERY, JOS, CLIN			0.43
	20	Surgery	PEN, AMO/CLA, OXA, CEF, LEV, ERY, JOS	8	4	1.03
	119	Pathology	PEN, AMO/CLA, OXA, CEF, KAN, TOB, LEV, JOS	7	2	1.22
	204	Respiratory	PEN, AMO/CLA, OXA, CEF, KAN, TOB, LEV, JOS	4	2	0.66
	2	Vascular	PEN, AMO/CLA, OXA, CEF, LEV, TEL, ERY, JOS	12	4	0.68
	174	Pathology	PEN, AMO/CLA, OXA, CEF, LEV, ERY, JOS, CLIN	4	2	1.51
	186	Extra-hospital	PEN, AMO/CLA, OXA, CEF, LEV, ERY, JOS, CLIN	4	4	1.08
	215	Unknown	PEN, AMO/CLA, OXA, CEF, KAN, TOB, LEV, TEL, ERY, JOS	5	4	1.21
	176	Vascular	PEN, AMO/CLA, OXA, CEF, GEN, KAN, TOB, LEV, ERY, JOS	12	8	1.24
	152	ICU	PEN, AMO/CLA, OXA, CEF, KAN, TOB, LEV, TEL, ERY, JOS, CLIN	4	3	1.14
	155	Respiratory	PEN, AMO/CLA, OXA, CEF, KAN, TOB, LEV, TEL, ERY, JOS, CLIN	8	6	0.53
	219	Pathology	PEN, AMO/CLA, OXA, CEF, GEN, KAN, TOB, LEV, TEL, ERY, JOS, CLIN	4	3	0.56
	104	Vascular	PEN, AMO/CLA, OXA, CEF, GEN, KAN, TOB, LEV, TEL, ERY, JOS, CLIN	8	4	1.40
	136	Surgery	PEN, AMO/CLA, OXA, CEF, GEN, KAN, TOB, LEV, TEL, ERY, JOS, CLIN	3	1	0.45
	94	Vascular	PEN, AMO/CLA, OXA, CEF, GEN, KAN, TOB, LEV, TEL, ERY, JOS, CLIN, COT, RIF	11	12	1.53
**G-12**	111	ICU	PEN, AMO/CLA, OXA, CEF, GEN, KAN, TOB, LEV, TEL, ERY, JOS, CLIN	16	8	1.48
	112	ICU	PEN, AMO/CLA, OXA, CEF, GEN, KAN, TOB, LEV, TEL, ERY, JOS, CLIN	8	3	0.50
**G-13**	13	Vascular	PEN	-	-	0.44
	49	Extra-hospital	PEN, COT	-	-	1.59
	14	ICU	PEN, KAN, TOB	8	4	0.68
**G-14**	32	Dermatology	No antibiotic resistance	-	-	0.60
	33	Dermatology	PEN, AMO/CLA, OXA, CEF, KAN, TOB, LEV, ERY, JOS	4	0.5	3.11
**G-15**	29	Vascular	ERY, JOS, COT	-	-	0.23
	30	ICU	PEN, AMO/CLA, OXA, CEF, GEN, KAN, TOB, LEV, TEL, ERY, JOS, CLIN	8	6	0.75

**G-16**	72	Surgery	No antibiotic resistance	-	-	0.60
	62	ICU	PEN, TEL	-	-	1.04
	53	Vascular	GEN, KAN, TOB, COT	8	4	1.26
	54	Respiratory	PEN, AMO/CLA, OXA, CEF, KAN, TOB, LEV, TEL, ERY, JOS, CLIN, COT	6	0.5	1.06
**G-17**	68	Miscellaneus	PEN, TEL	6	4	1.01
**G-18**	55	Unknown	PEN	-	-	0.71
	73	Surgery	PEN	-	-	0.80
	105	Unknown	PEN, TEL	12	8	0.66
**G-19**	48	Extra-hospi-tal	PEN	8	12	2.79
**G-20**	35	Unknown	PEN	-	-	0.37
	47	Vascular	PEN	8	0.5	1.51
	52	Vascular	PEN	-	-	1.50
**G-21**	75	Miscellaneus	PEN, KAN, TOB, TEL, ERY, JOS	9	8	0.64

In shadow, the MRSA strains.
