# Supplementary figures and images for: Antimicrobial Activity of the Circular Bacteriocin AS-48 against Clinical Multidrug-Resistant Staphylococcus aureus
Source: Antibiotics (Basel). 2021 Jul 30;10(8):925. doi: 10.3390/antibiotics10080925 (PMC8388780; doi:10.3390/antibiotics10080925)

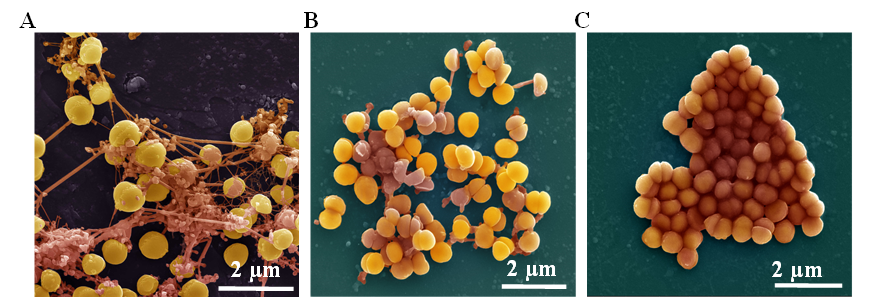

Supplement: Supplementary file 1 [file antibiotics-10-00925-s001.zip › antibiotics-1285030-supplementary/antibiotics-1285030-supplementary-final/Supplementary Figure 1.tif]
